# Modern Sociology

**Published:** 1896-10-03

**Authors:** 


### Modern Sociology.

### THE HELLENIC AND GREEK INFLUENCE.

The late Sir Henry Maine, in one of the most famous of his lectures, described the ancient Greeks as the only nation of that period of the world's history who understood the idea and meaning of "progress." On another occasion he called attention to the fact that the idea of "progress" was utterly alien to the greater portion of mankind, and especially so to the vast populations of the East in India and China, where "caste" and "custom" have reigned supreme for countless ages. Few will be found to dispute Sir Henry Maine's judgment as far as art, literature, science, and philosopy are concerned; but in the domain of morality and religion the question is more doubtful, although it must be admitted that these subjects have never been treated with greater sublimity than by Plato, and with a greater regard to their utilitarian aspects than by Aristotle.

From a sociological point of view, in studying the communities of ancient times, one great and all-important fact must be constantly borne in mind—the universal prevalence of slavery. To the Jew all other races of men were Gentiles, and to the Greek, barbarians, intrinsically inferior to himself in every way. Therefore any sociology in the modern sense of the word was absolutely impossible in the ancient world. It is true that occasionally we get some faint glimmerings of a wider view of humanity in the works of the poets and the philosophers, and the philosophical Emperor, Marcus Aurelius, who wrote his great work in the Greek language, quoted passages from the works of Epictetus, a deformed slave; but this was after the light of Christianity had begun to dawn upon the world, and, perhaps, when it was already beginning to exert its beneficent influence. A famous preacher once said, that "Nations are the creations of God working in history," and an equally famous historian has remarked that nations, like individuals, sometimes go wrong. It is this which gives a peculiar interest to the study of the history of the great ancient nations who, though dead politically, yet speak to us of the modern world. If we take the picture of the Society of Athens, as depicted by her greatest statesman, Pericles, as embodying the highest ideal of Hellenic life we shall form a tolerably true conception of what the ancient Greeks considered as the ideal life of a citizen. "We live under a constitution such as in no way to envy the laws of our neighbours—ourselves an example to others, rather than mere imitators. It is called a democracy, since its permanent aim tends towards the many, and not towards the few. As to private matters and disputes, the laws deal equally with every man; while in regard to public affairs and to claims of individual influence, every man's chance of advancement is determined not by party favour, but by real worth, according as his reputation stands in his own particular department. Neither poverty nor obscure station keep him back, if he really has the means of benefiting the city. Moreover, our social march is free, not merely in regard to public affairs, but also in regard to intolerance of each other's diversity of daily pursuits. For we are not angry with our neighbour for what he may do to please himself, nor do we ever put on those 'sour looks' which, though they do no positive damage, are not the less sure to offend." This passage shows very clearly the Greek conception of both public and private life, and is worthy of the closest attention by all who wish to compare the life of the old world, which has so profoundly influenced and modified our own civilisation, with that of the societies of modern times, whether they be aristocratical, democratical, or monarchical. It is an ideal which has certainly never been yet attained to by any modern society, and as such is of extreme interest to the student of sociology—a subject which must always be treated rather by the results of experience than by those of experiment. But with all the joyous vivacity and versatility of the ancient Greek life, with all its public games and festivals, there was always a sombre fatalism in the background. We speak of a man "meeting" his fate. To the Greek mind, a man endeavours to escape from his fate, which is always pursuing him. In the "Odyssey," Achilles says to Ulysses, when he meets him in the lower world, that he would rather be a herdsman of cattle in his earthly father's realm than a great prince among the Shades. When the Greek States lost their freedom, and, under the rule of a non-Hellenic conqueror—Alexander the Great—conquered the Eastern world, then Greek influence began to make itself really felt far beyond the boundaries of Greece proper. A still greater triumph was in store for the Greeks, after they had fallen under the political dominion of Rome. The conquered took the conqueror captive. There was, so to speak, an "international marriage," on terms of equality, between Greece and Rome. The West was taken captive, as well as the East. As a great historian of Rome has well said, "The higher Roman culture itself was, in fact, nothing else than the proclamation of the great gospel of Hellenic manners and art in the Italian idiom." The Greek and the Latin languages were taught together in all the great schools and universities of the Roman Empire, which was then almost coterminous with the civilized world. Later on the seat of Empire was itself transferred to a Greek city on the shores of the Bosphorus, and ultimately the Empire itself became Greek. A nation with such a wonderful history as that of the various Hellenic States cannot fail to have left a deep and abiding impression upon all other races which came into contact with it. And since it became the teacher of its great conqueror, Rome, upon the ruins of which universal Empire the chief states of Western Europe have been built, we may say that its influence is still upon us, for to use the words of our own great English historian, "the roots of the present lie buried away deep in the past." Until the great discoveries in the various fields of natural science, which have distinguished the nineteenth century from all others, human knowledge had advanced very little, if at all, from the point at which it was left by the Greeks. Curiously enough, this branch of knowledge was almost untouched by them, as Socrates considered that the "proper study of mankind was man," and that the secrets of physical and natural science were beyond human comprehension. In the present day, there seems to be a reaction in favour of the old Greek theory; and the study of sociology is one of the most marked symptoms of this reaction.

